# Bulletin of the Buffalo General Hospital

**Published:** 1912-01

**Authors:** 


**Bulletin of the Buffalo General Hospital**

VOL. I JANUARY, 1912 No. 3

### Anaesthesia by Intra-Tracheal Insufflation

### The New Apparatus at the Surgical Clinic of the General Hospital

THE question of anesthetics and of anesthesia is by no means yet an absolutely settled one, but the conviction is constantly gaining ground that as between the two, the man is more important than the anesthetic which he may administer; in other words, given by a reliable man, any of the anaesthetics in common use may be safely administered,—without the proper man not one of them. Next to the man, however, comes not merely the selection of the drug itself, but of the method of its administration. There certainly is a large choice as between methods. Beyond the mere question of method there stand also the mechanics and the mechanism of respiration. These topics are of surpassing interest when it becomes a question of opening the chest with the greatest degree of safety. To Dr. Meltzer, of New York, belongs the greatest share of credit for practically demonstrating that in man all that is required for satisfactory respiration is that a constant current of air shall be delivered at or near the division of the wind-pipe under an exceedingly gentle pressure; and that if this air be medicated with a certain percentage of ether vapor, anesthesia is rapidly produced and safely continued. If air be delivered, i.e., pumped in, under these conditions, there is no need for respiratory movement on the part of the patient; everything is done for him. Oxygen is delivered at the gateway of his capillaries just as pre-digested food can be delivered within the stomach, virtually all ready for use. Under these circumstances both life and anesthesia may be safely maintained by a simple, mechanical contrivance, for as many hours as may be desired.

Such an apparatus has just been placed in operation in the surgical clinic at the General Hospital. It consists of a little pump run by a motor which passes the air through a receptacle containing ether, this being provided with such an arrangement that exactly the desired strength of medication with ether-vapor can be automatically maintained; this is connected with a small tube which is passed down the trachea, and halted at about one inch above its bifurcation. The motor once in operation, air is delivered under a few millimeters pressure, which is just sufficient to carry it to the remotest air cells, and there effect the oxygen interchange. The return current passes upward and backward along the trachea, which has been only partially occluded by the tube. It is a wonderful sight to see the purpose of respiration accomplished so satisfactorily without requiring any movement on the part of the patient's chest-wall, his breathing being thus done for him. By this new expedient the former questions, which so terribly complicated thoracic surgery, of negative and positive pressure, have been entirely superseded. When necessary, one or both lungs, and even the heart itself, may be widely exposed, were it necessary, for hours, while after complete closure of the chest natural respiration is resumed with a minimum of post-operative discomfort. The apparatus is that designed by Dr. Charles Elsberg, and while it is not necessary to resort to it in ordinary cases, in which none of the extraordinary problems of anesthesia are presented, it is of the greatest importance to have it at hand in cases of neck and thoracic surgery, while in cases of opium poisoning or suspended animation, it supplies the method and the means of continuing artificial respiration indefinitely.

---

### The Nurses Loan Fund

THE General Hospital has been more fortunate than many institutions of its class in securing good material for its Training School, but from time to time desirable applicants are lost, because of the long term during which a pupil nurse can earn nothing. Slight though those expenses may be, relatively speaking, for which the Hospital does not provide during the three years of training, they are sometimes more than the would-be nurse can meet. The Trustees have accordingly established a Loan Fund, to be administered under the following conditions:

First: Such loans shall not exceed an aggregate amount of $75.00 to any one person, and shall be granted only to such as have proved themselves worthy and who have been in the school for a period of not less than one year. In this connection, however, it is further recommended that should instances arise wherein, because of unusual circumstances, it is deemed advisable to advance funds to a pupil before the expiration of the first year of work, the President shall act at his discretion.

Second: Such loans shall be covered by a demand note in favor of the Hospital, bearing 3 per cent. interest from date, but with the understanding that no call for payment shall be made before the first year after graduation.

Third: These loans shall be made on the recommendation of the Superintendent of Nurses, subject to the approval of the President.

Fourth: The fund and all notes covering loans from it shall be in the care of the Superintendent of the Hospital, and a proper account be kept of it.

The fund amounts to about $600.00.

---

The Trustees have authorized the Visiting Committee of the Trustees to associate with them others, from outside the Board of Trustees, who are especially interested in hospital work, and to form such sub-committees as seem needed. One such committee will assist in the Social Service Work of the Hospital, and another may have special charge of the Harrington. It is also provided that the Trustees shall be represented by one of their number on each of these committees.

---

### Christmas at the Hospital

The most beautiful feature of Christmas at the Hospital is the singing of the carols by the Trinity Church choir on Christmas Eve. The doors leading into the patients' quarters are everywhere opened; the choir, starting near the center of the building, comes slowly down the corridor, singing as they come, and halts at the entrance of each ward in turn. With the changing hospital population, each year finds a body of patients to whom the event is entirely unexpected, and to see the Christmas spirit at its best one need but stand at the door of a ward and watch the effect as the first faint notes are heard —the languid interest, then the lifted head and the quickened wonder and delight as the song grows in volume, the choristers halt at the ward door, and finally the notes die in the distance.

The Hospital was, as always, effectively decorated, through the generosity of its friends, who also provided the Christmas dinner. The peculiar digestive powers developed by even the most disabled patients in the face of its temptations, have never been given the scientific attention which the phenomenon deserves.

The children had their tree—this year in their own hospital. There is much skepticism in those wards: "He'll never come. I've watched the door all the time." But he always does.

---

During the winter months religious services are held at the Hospital twice a month. On one Sunday the service is held in Scatcherd Chapel, under the auspices of the Laymeh's League; the other service, like that of Christmas Eve, is held at the wards, to which the minister and singers go in turn. There is a brief reading, a prayer, and music, bringing that solace to those who must otherwise be deprived of it.

---

### The Social Service Work

THE Social Service Department, instituted last January, presented its first summarizing report at the end of the Hospital's fiscal year, October 1, and was able to make a notable showing. The department itself is small, consisting of a head worker, one assistant and a substitute, and eight volunteers; but they have drawn into co-operation with them fifty-three associations, charitable agencies and public officials. One of the most interesting developments has been the appointment of a committee by the Polish Charity Circle, to act as interpreters for Polish patients. They have been of great service in securing the histories needed by the social workers. The report of this department, which is given in detail in the annual report of the Hospital, is to be commended to the attention not only of humanitarians, but students of social conditions as well. It gives data of significance. Sixteen nationalities were represented in the list of beneficiaries, seven of them furnishing but one case each. On the other hand, one leading immigrant people, who had sixty-three patients in the Hospital during the year, furnished thirty-two cases to the social workers. The two thousand six hundred and thirty-one Americans, in the same time, furnished seventy, the two hundred and thirteen Germans but four. But a more interesting comparison is between this first immigrant group and another, which, in the year, had seventy-nine representatives at the Hospital, but five of whom required a helping hand from the social workers. Are we to infer that the first group is disproportionately "unfortunate" or lacking in thrift? And is the second, which, in proportion to its numbers in the city, provides many more hospital patients than the first group, a stock especially lacking in physical vigor? Whatever an adequate study of these questions might show, we already see that the social service work, while assisting the individual, is also gathering data of social importance. This is a function of the hospital just beginning to be recognized, and it promises a by-product of high value.

